# Assessment of antioxidant and antibacterial efficacy of some indigenous vegetables consumed by the Manipuri community in Sylhet, Bangladesh

**DOI:** 10.1016/j.heliyon.2024.e37750

**Published:** 2024-09-12

**Authors:** Mukta Roy, Jahid Hasan Shourove, Rhythm Singha, Tawkir Ahmed Tonmoy, Gokul Chandra Biswas, Fariha Chowdhury Meem, Parvej Hasan John, Mitu Samadder, Md. Azmain Al Faik

**Affiliations:** a Food Engineering and Tea Technology, Shahjalal University of Science and Technology, Sylhet, Bangladesh; b Genetic Engineering and Biotechnology, Shahjalal University of Science and Technology, Sylhet, Bangladesh

**Keywords:** Indigenous, Traditional food, IC_50_ value, Total flavonoid content, Minimum inhibitory concentration, Minimum bactericidal concentration

## Abstract

The rapid dietary changes experienced by indigenous people worldwide threaten the use of traditional foods, which are often undervalued. This study focused on evaluating the antioxidant and antibacterial efficacy of five vegetables typically consumed by the Manipuri ethnic groups in the Sylhet region of Bangladesh: Yongchak seed (*Parkia speciosa*), Telikadam seed (*Leucaena leucocephala*), Phakphai leaf (*Persicaria odorata*), Sheuli leaf (*Nyctanthes arbor-tristis*), and bamboo shoot (*Bambusa* spp.). The samples were dried and powdered to assess the antioxidant activity through total phenolic content (TPC), total flavonoid content (TFC), total tannin content (TTC), and 2,2-diphenyl-1-picrylhydrazyl (DPPH) radical scavenging activity. Antibacterial efficacy was determined by measuring the zone of inhibition (ZOI), minimum inhibitory concentration (MIC), and minimum bactericidal concentration (MBC). Leafy vegetables exhibited higher TPC, TFC, and TTC than seeds and shoots, with *N. arbor-tristis* leaf showing the highest TPC (99.16 ± 2.07 mg GAE/g DW) and *P. odorata* leaf exhibiting the highest TFC (9.19 ± 0.7 mg QE/g) and TTC (3.59 ± 0.26 mg TAE/g). However, *Bambusa* spp. shoot extract showed the highest antioxidant potential (IC_50_: 1.66 ± 0.05 mg/mL). All samples exhibited higher ZOI against gram-positive bacteria (*Bacillus* spp. and *Staphylococcus* spp.), ranging from 10 ± 2.65 to 19.33 ± 2.08 mm. *L. leucocephala* seed extract showed the highest antibacterial activity against both the tested gram-positive bacteria with a MIC of 15.6 mg/mL. Conversely, the *P. odorata* leaf extract exerted the strongest antibacterial effect against gram-negative bacteria, with the lowest MIC values for *Klebsiella* spp. (31.25 mg/mL) and *Escheria coli* (62.5 mg/mL). The findings of this investigation suggest that the selected indigenous vegetables could be valuable sources of phytochemicals with potential antioxidant and antibacterial activities. Incorporating and promoting these traditional foods into the diet may improve food security, dietary diversity, and public health in Bangladesh.

## Highlights

• The selected Manipuri vegetables are good sources of natural antioxidants and antibacterials.
• Phenolic compounds were comparatively higher in leafy vegetables than shoots and seeds.
• Bamboo shoot extract exhibited the highest antioxidant potential (IC_50_ = 1.66 ± 0.05 mg/mL).
• The tested gram-positive bacterial strains were more sensitive to the sample extracts than the gram-negative ones.
• The *Persicaria odorata* leaf extract exhibited great antibacterial potency against gram-negative bacteria.

## Graphical abstract

**Figure:**
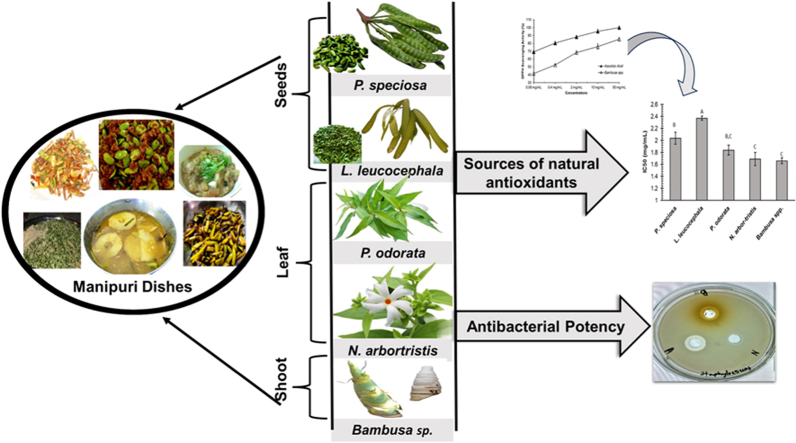

## 1 Introduction

Plant-based ethnic foods are rich in nutrients and valuable chemical compounds, making them a potential source for developing medicines [1]. In particular, the antioxidant and antibacterial capacities of ethnic foods have grabbed attention for their prospective health benefits [2]. The presence of numerous phytochemicals in plant-based foods, including phenolics, flavonoids, steroids, saponins, alkaloids, and glycosides, supports biological processes in the human body [3,4]. Phenolics and flavonoids are especially notable for their hydroxyl groups, which allow them to deactivate singlet and triplet oxygen, neutralize free radicals, and degrade peroxides by contributing a hydrogen atom or an electron [5]. Oxidative stress, resulting from an imbalance between the generation of free radicals and the body's capacity to neutralize their detrimental effects, has been implicated in various chronic illnesses, such as cancer, autoimmune disease, cardiac and neurodegenerative disorders [6]. Phytochemicals work as natural antioxidants and protect the body from oxidative stress by counteracting harmful free radicals [7]. Furthermore, studies have indicated that fruits and vegetables rich in polyphenols demonstrate promising thrombolytic activity [[8], [9], [10]].

Nowadays, antibiotic resistance against synthetic antibiotics and antibacterial agents is becoming a concern for public health, resulting from their indiscriminate usage [[11], [12], [13]]. In that regard, medicinal plants have served as the cornerstone of alternative medicine and a mainstay in the process of developing new pharmaceuticals [14]. Plant extracts can exhibit antibacterial activity as they contain several bioactive compounds, including alkaloids, organic acids [15], flavonoids [16], terpenoids, and phenolic compounds. These compounds can inhibit bacterial growth by disrupting their cell membrane, interfering with DNA replication or protein synthesis, and causing oxidative stress [17]. Numerous studies have proven that plant extracts are highly effective in combating the microorganisms responsible for food poisoning [4,[18], [19], [20], [21], [22]]. Therefore, it is crucial to thoroughly investigate plants that possess a significant amount of phenolic and flavonoid compounds.

Tribal societies around the world have historically had a deep understanding of the native plants and other natural resources that they directly depend on [23]. To preserve and gather invaluable traditional knowledge regarding native food plants and raise awareness of their significance, extensive research studies are necessary [24]. Traditional knowledge of local food plants is essential to utilizing this great food source for the local population and can help achieve the sustainable development goal (SDG) of zero-hunger. There is a discrepancy in the distribution of food insecurity and the negative health consequences it brings, with higher rates among ethnic minority groups. SDG targets two, “Zero Hunger,” and fifteen, “Life on Land,” emphasize the necessity of local stakeholders’ participation to achieve the goals of preserving biodiversity, ensuring food security, and halting land degradation [25]. With rising population demand, traditional and ethnic foods will play a greater role in individual dietary patterns. Since they are regarded as nutritious foods, demand for them has increased significantly [26]. Thus, additional research on the nutrient composition and health benefits of plan-based ethnic foods may result in the creation of new crops to satisfy current consumer demands. While the scientific literature has extensively investigated the antioxidant and antibacterial properties of various food sources, there is limited research explicitly focused on the indigenous food plants used by the Manipuri community in Sylhet. According to a recent survey conducted by the Bangladesh Rural Advancement Committee (BRAC), more than 70 indigenous communities exist in Bangladesh [27]. In Sylhet, the Manipuri is one of the leading indigenous groups, with a population of 139,000 [28]. The Manipuri community living in the greater Sylhet region has a rich culinary heritage that includes the utilization of various indigenous food plants in their traditional cuisine [23]. They prefer raw-flavored foods and tend to avoid processed food items. In any community, the development of habitual features is greatly influenced by geo-topographical elements. Since they are accustomed to cultivating their own vegetables, the majority of their diet consists of plant-based foods. They grow some unique vegetables that are not commonly found in other places in Bangladesh [29].

Among the Manipuri foods, the Stink bean (*Parkia speciosa* Hassk.) is a popular vegetable commonly known as Yongchaak [30]. The seeds or the entire bean of *Parkia* are consumed by incorporating them into a traditional dish known as Eromba or Yongchaak singju (salad) or in the form of pickles. Eromba is a famous cuisine among Manipuri, prepared by combining boiled potato, fermented fish, chili, and various vegetables, including *Parkia*. Yongchaak singju, on the other hand, is a beloved side dish that involves cutting *Parkia* into small pieces and mixing it with a spicy red chili paste [30,31].

Phakphai (*Persicaria odorata* (Lour.) Soják) is another leafy vegetable known for its distinctive flavor, and its leaf is utilized as a key ingredient in various Manipuri as well as other Southeast Asian dishes, including soups, stews, curries, and salads [32,33]. Furthermore, this plant is widely recognized for its culinary and medicinal uses [32]. Lead tree (*Leucaena leucocephala* (Lam.) de Wit) seed is also a popular vegetable used as a fresh side dish by Manipuri people in Bangladesh and Thailand [34]. It is commonly known as telikadam seed among Manipuri people [35]. Telikadam seed is famous for its phytochemicals having medicinal value in treating stomach ailments, facilitating abortion, and uterine contraction, diabetes [36]. Night-flowering jasmine (*Nyctanthes arbor-tristis* L.), commonly known as “sheuli” in the local language, is also renowned for its traditional medicinal properties. Sheuli is a widely consumed leafy vegetable among Manipuri people [37]. The fresh leaves of *Nyctanthes* are also used to prepare juice, which is consumed as a traditional remedy for various health conditions such as persistent fevers, malaria, rheumatic fever, hepatic diseases, and constipation in children [38]. Bamboo shoot is a widely consumed Manipuri vegetable, imparting distinct flavors to traditional dishes and pickles. Shoots of *Bambusa* spp. contain only 27 % of edible parts [39]. All these foods are traditionally consumed by the Manipuri people and used in several disease treatments.

Therefore, assessing the antioxidant and antibacterial efficacy of these indigenous vegetables is necessary to gain a more comprehensive understanding of their potential health benefits. This study focuses on the assessment of the antioxidant and antibacterial capacities of selected indigenous food plants used by the Manipuri community. To the best of our knowledge, this is the first study on these Manipuri vegetables grown in the Sylhet region, Bangladesh. Exploring the knowledge of these ethnic foods will significantly contribute to the preservation and sustainability of traditional food systems and cultures. Moreover, research on these indigenous vegetables can provide valuable insights so that the vegetables can be incorporated into diets across the country. In a broader sense, the findings of this study may contribute to the National Food and Nutrition Security Policy (NFNSP) undertaken by the Government of Bangladesh, which aims to guarantee that the country achieves its nutritional and food security-related SDGs, as well as fulfills the national and international commitments by 2030 [40].

## 2 Materials and methods

### 2.1 Chemicals and reagents

The aluminum-trichloride, Folin-Ciocalteu reagent, 2,2-Diphenyl-1-picrylhydrazyl (DPPH), methanol, potassium-acetate, sodium carbonate, sodium hydroxide, standard gallic acid, standard ascorbic acid, tannic acid, and quercetin were purchased from Merck, Germany. Mueller Hinton agar and nutrient broth media were supplied by Hi-Media, Mumbai. The standard antibiotic (azithromycin) was provided by ACME Laboratories Ltd., Bangladesh. All the other chemicals employed in this research were of analytical grade.

### 2.2 Sample collection

**Fig. 1:**
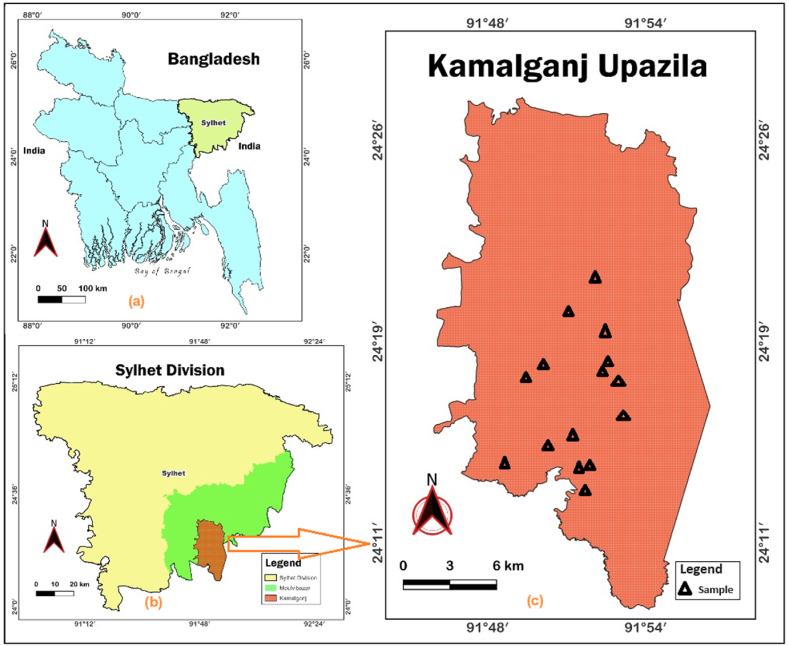
Location of sampling sites: (a) on the map of Bangladesh, (b) extended view of Sylhet division, and (c) an extended view of Kamalganj upazila indicating the positions of fifteen sampling fields where samples were collected.

Five different types of commonly consumed plant foods including stink bean/Yongchak seed (*P. speciosa*), Telikadam seed (*L. leucocephala*), Phakphai leaf (*P. odorata*), Shiuly flower/night flowering jasmine leaf (*N. arbor-tristis*), and Bashkorol/bamboo shoot (*Bambusa* spp.) were collected from the Indigenous community (Bishnupriya, Manipuri) lived in Kamalgonj upazila (24°20′N, 91°51′E) of the Moulvibazar district in the division of Sylhet, Bangladesh. Fresh samples, approximately 2 kg each, were collected from at least three different sampling sites. The samples were placed in zipper bags and transported to the laboratory for further analysis. The sample selection sites are represented in Fig. 1.

### 2.3 Preparation of sample

**Fig. 2:**
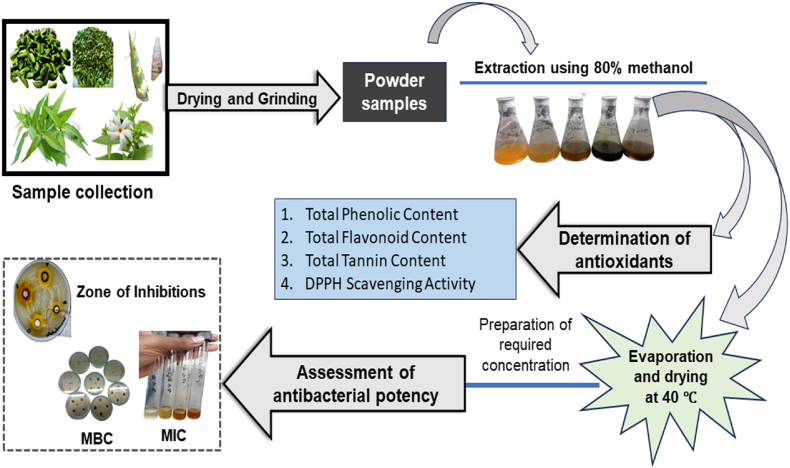
Schematic diagram of research design.

The collected samples were washed with fresh water and then chopped into smaller pieces. Afterward, they were dried at 60 °C for 48 h, and ground into fine powder using a grinding machine (3390D40, Thomas Scientific, USA). The powdered samples were passed through a sieve (24 mesh) and collected into an airtight container for further use. This study took place in the research facility of the “Department of Food Engineering and Tea Technology and the Department of Genetic Engineering, Shahjalal University of Science and Technology, Sylhet-3114, Bangladesh”. The complete research framework for this study is illustrated in Fig. 2.

#### 2.3.1 Sample extraction for antioxidants

The powdered sample (5 g) was taken and mixed with 50 mL of 80 % methanol. This mixture was then placed in a shaking incubator (SI-100, HUMAN Lab, Korea) at 25 °C for 3 h at 150 rpm. After shaking, the crude extract was centrifuged at 4,000 rpm at a centrifuge machine (416G, Gyrozen, Korea) for 10 min. Then, the solution was filtered with Whatman filter paper (No. 1). The aliquots were collected and stored at − 20 °C for further analyses [41].

#### 2.3.2 Sample extraction for the assessment of antibacterial activity

The finely powdered sample (100 g) was soaked in 200 mL of 80 % methanol and shaken in the shaking incubator for 24 h. They were then filtered using the Whatman filter paper (No. 1). The resulting clear filtrates were evaporated and dried at 40 °C with a rotary vacuum evaporator (HS-2005S, HANSHIN, Korea), followed by freeze-drying using a freeze dryer (LyoQuest-55, Telstar, Spain). Finally, the dry extract powder (200 mg) was weighed and dissolved in 0.4 mL of methanol to achieve a concentration of 500 mg/mL. The obtained stock solutions were stored at − 20 °C for testing antibacterial activity [42].

### 2.4 Assessment of total phenolic content (TPC)

The TPC in the selected Manipuri vegetables was measured according to a modified Folin-Ciocalteu method [43]. Briefly, 20 μL of each sample extract was taken into test tube, followed by the addition of distilled water (1.58 mL) and Folin-Ciocalteu reagent (100 μL). Then, they were well shaken, and sodium carbonate (20 %) was added in the amount of 300 μL within 8 min. The resultant mixture was subjected to vortexing and kept for 30 min at 40 °C maintaining dark conditions. The absorbance was recorded at 765 nm using a UV–Vis spectrophotometer (Model-UV-1800, Shimadzu, Japan). A calibration-curve was prepared by repeating the same procedure with gallic acid solutions at concentrations 0–50 mg/L. The TPC was calculated from the following equation: Y = 0.9028x + 0.054, R^2^ = 0.9998, whereas Y is the absorbance and x is the concentration of phenolic compounds expressed in mg equivalents of gallic acid per gram of dry weight (mg GAE/g DW).

### 2.5 Assessment of total flavonoid content (TFC)

The TFC in the selected Manipuri vegetables was assessed according to the aluminum trichloride method [44]. Firstly, 0.5 mL of the sample extract was taken in a test tube. Then, 1.5 mL ethanol (95 %), 2.8 mL of distilled water, 0.1 mL of potassium-acetate (1 M), and 0.1 mL of aluminum-trichloride (10 %) were added into the test tube and stood for 40 min at room temperature. Finally, the absorbance of the mixture was recorded at 415 nm using a UV–Vis spectrophotometer against a distilled water blank. A calibration curve was constructed using standard quercetin in the concentration range of 20–100 mg/L. The TFC was calculated from the following equation of the quercetin standard curve: Y = 0.004x + 0.0236, R^2^ = 0.998, whereas Y is the absorbance and x is the concentration of flavonoid compounds expressed as mg equivalents of quercetin per gram of dry weight (mg QE/g DW).

### 2.6 Assessment of total tannin content (TTC)

The TTC was quantified employing a modified Folin-Ciocalteu method, as delineated by Haile and Kang [45,46]. The analysis was performed by adding 0.1 mL of plant extract with 0.5 mL of Folin-Ciocalteu reagent and 7.5 mL of distilled water. The mixture was allowed to sit at room temperature for 5 min. Subsequently, 1 mL of 35 % sodium carbonate was introduced into the solution, followed by the addition of distilled water to adjust the final volume to 10 mL. After shaking and standing for 30 min at room temperature, the absorbance of the resulting mixture was recorded at 700 nm using a UV–Vis spectrophotometer. A blank sample was prepared using distilled water in place of the sample extracts. A set of standard solutions of tannic acid (ranging from 0 to 100 mg/L) was read against the blank. The TTC was calculated from the following equation of the tannic acid standard curve: Y = 0.0015x + 0.022, R^2^ = 0.997, whereas Y is the absorbance, and x is the concentration of tannin compounds expressed as mg equivalents of tannic acid per gram of dry weight (mg TAE/g DW).

### 2.7 Assessment of 2,2-diphenyl-1-picrylhydrazyl (DPPH) scavenging activity

The radical scavenging activity of the vegetable samples was measured by following the method of Brand-Williams et al. [47]. In this analysis, sample extracts were serially diluted five-fold to yield concentrations of 0.08, 0.40, 2, 10, and 50 mg/mL in methanol. Then, 1 mL of the methanolic extracts was mixed with 0.15 mM DPPH solution (2 mL) and it was kept in dark for 20 min. Finally, using a UV–Vis spectrophotometer, the absorbance at 517 nm was recorded against blank (1 mL 80 % methanol mixed with 2 mL of DPPH radical solution). The radical scavenging activity (%) was calculated as follows:

$$Radical\;scavenging\;activity\;\left(\%\right)=\frac{A_{0}-A_{s}}{A_{0}}\times 100$$

where A_0_ is the absorbance of the control blank and A_s_ is the absorbance of the sample extract. The IC_50_ value, which denotes the concentration needed to neutralize 50 % of DPPH radicals, was calculated from the plot illustrating scavenging activity against extract concentration.

### 2.8 Inoculum preparation

The inoculum preparation was accomplished by using the technique followed by Atef et al. [42]. The test organisms were obtained from the “Department of Genetic Engineering and Biotechnology, Shahjalal University of Science and Technology”. They include 2 g-positive bacteria (*Staphylococcus* spp. and *Bacillus* spp.) and 2 g-negative (*Klebsiella* spp. *and Escherichia coli*), all of which are human pathogens. For the standardization of inoculum, nutrient broth (5.0 mL) was taken where a loopful of the selected bacterial strain was inoculated and cultured at 37 °C for 24 h. Subsequently, 0.2 mL of the culture was transferred to 20 mL of nutrient broth, following the incubation period of 3–5 h to achieve the desired density of 10^6^ colony-forming units (CFU)/mL.

### 2.9 Determination of zone of inhibition (ZOI)

The ZOI was measured by the agar well diffusion technique as outlined by Atef et al. [42]. Briefly, 100 μL of bacterial culture (10^6^ CFU/mL) was inoculated on a Mueller Hinton agar plate using a sterile swab. The solidified agar medium was punched with a cork-borer to prepare a well of 6 mm diameter. Then, 100 μL of plant extract having a concentration of 500 mg/mL was placed into the wells. Azithromycin (30 μg/mL) was used as a positive control, while methanol served as the negative control. After allowing the plates to stand for 1 h to facilitate the pre-diffusion of the extracts, they were incubated at 37 ± 2 °C for 24–48 h under controlled aerobic conditions. Finally, the ZOI was measured in millimeters (mm).

### 2.10 Determination of minimum inhibitory concentration (MIC) and minimum bactericidal concentration (MBC)

The MIC value of the samples was measured using the micro-broth dilution technique followed by Atef et al. [42]. Firstly, 100 μL of sterile nutrient broth was taken in each test tube. Subsequently, 100 μL of prepared extract (500 mg/mL) was put into the first test tube. A final concentration of 250 mg/mL was obtained in the first test tube by this dilution process. Then it was subjected to two-fold serial dilutions by transferring 100 μL from the first tube into the next one, resulting in 125, 62.5, 31.25, 15.62, and 7.81 mg/mL concentrations, respectively. Each test tube was inoculated with 100 μL of a bacterial solution containing 10^6^ CFU/mL and mixed gently by shaking. The tubes were then incubated for 24 h at 37 °C. Based on the turbidity that resulted in the test tube, the MIC was determined to be the lowest concentration of the extract that inhibited bacterial growth.

After the MIC determination, further growth of bacteria was observed on the nutrient agar plate under inoculation of a loopful of the bacteria and sample extract mixture from all the tubes that showed no visible growth in the MIC test. The incubation temperature was kept at 37 °C for a time period of 24 h. The lowest concentration of sample extracts where no bacterial growth was observed was considered as the MBC, confirmed by comparing bacterial growth on agar plates before and after incubation.

### 2.11 Statistical analysis

The experiments were repeated three times and the data analysis was performed using the STATA version 17. One-way ANOVA using Tukey's method was performed to measure significant differences among the results when p < 0.05. The results were provided as the mean ± standard deviation.

## 3 Results and discussion

### 3.1 Total phenolic content

**Fig. 3:**
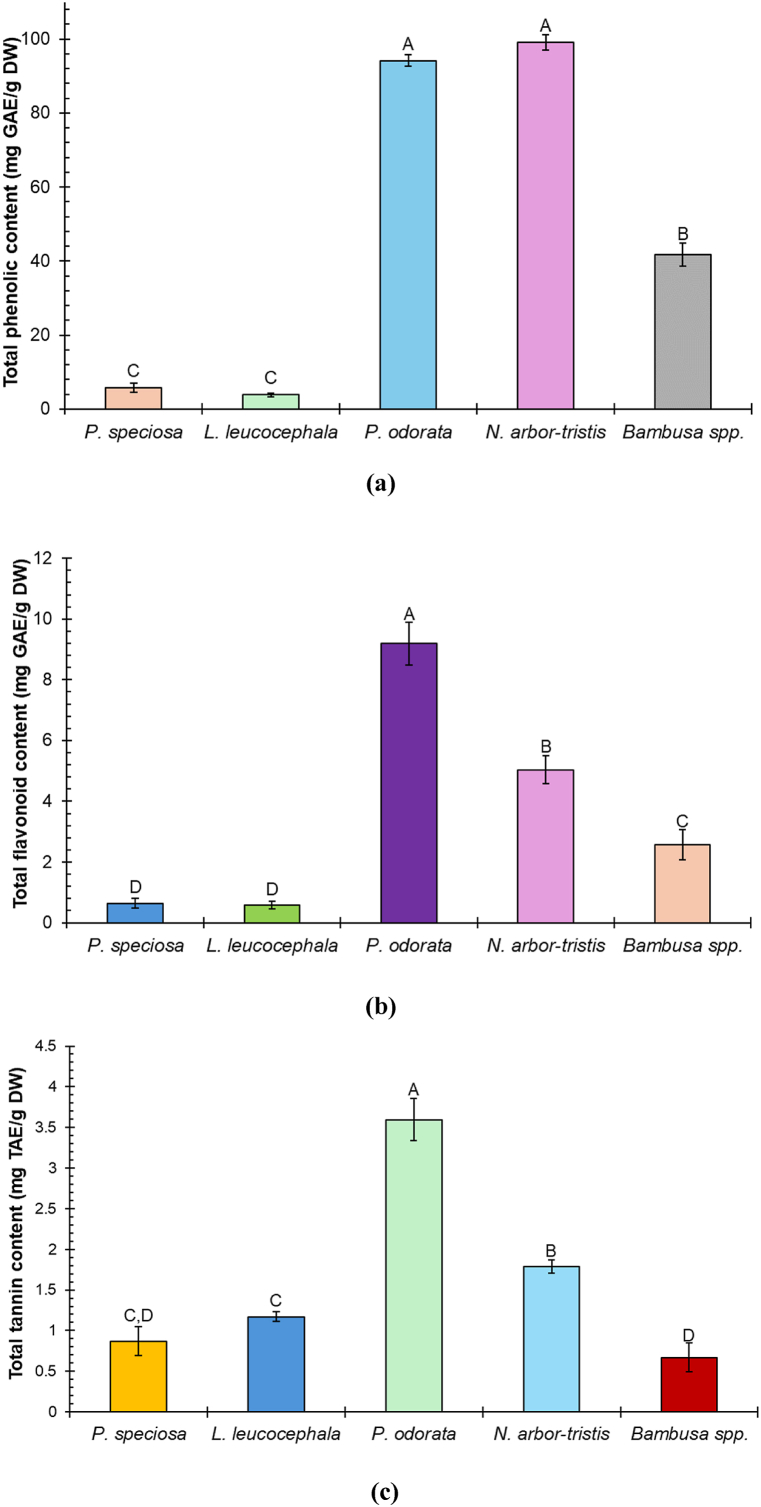
(a) Total phenolic content; (b) Total flavonoid content, and (c) Total tannin content of five Manipuri vegetables. Results represent means ± standard deviation (n = 3). Different letters in the column indicate the significant differences among the samples (Tukey's test, p < 0.05).

This study examined five indigenous vegetables commonly used in Manipuri delicacies as well as Southeast Asian cuisines. The TPC of the selected Manipuri vegetables is shown in Fig. 3a. The TPC values decreased in the following order: *N. arbor-tristis* leaf > *P. odorata* leaf > *Bambusa* spp. shoot > *P. speciosa* seed > *L. leucocephala* seed.

Phenolic compounds act as powerful *in vitro* antioxidants by providing hydrogen or electrons, thereby stabilizing free radicals [48]. This study revealed that the TPC was significantly higher (p < 0.05) in leafy vegetables such as *N. arbor-tristis* (99.16 ± 2.07 mg GAE/g DW) and *P. odorata* (94.151 ± 1.6 mg GAE/g DW) compared to the seeds and shoots examined (Fig. 3a). A previous study on the methanolic extract of *P. odorata* leaves reported 52.59 mg GAE/g DW, which is significantly lower than our findings [49]. Another study found a lower TPC in the ethanolic extract of *P. odorata* leaves (58.56 ± 3.86 μg GAE/mg DW) [50]. However, a higher TPC (137.5 ± 1.28 mg GAE/g DW) was observed in the ethanolic extract of *P. odorata* leaves in Malaysia [51]. These variations in TPC may result from the solvent used in the extraction process, extraction condition, plant variety, soil condition, and other climatic factors [52]. In a previous study, genetic diversity was found to be associated with the variation in TPC among nettle leaves [41]. A recent study by Gahtori et al. reported a TPC of 63.50 mg/mL in the ethanolic extract of *N. arbortristis* leaves [38], which is lower than the TPC observed in our study. Another study on *N. arbor-tristis* leaves indicated that TPC levels varied with the solvents used and ranged from 159.50 to 201.00 μg GAE/mg DW [53]. The high TPC in *N. arbor-tristis* is attributed to the presence of protocatechuic acid, chlorogenic acid, caffeic acid, and gallic acid [54]. The TPC of the two leafy vegetables we studied was higher than that of the previously studied commonly consumed leafy vegetables such as moringa leaves *(Moringa oleifera*: 64.6 ± 0.34 mg GAE/g DW) [46], and water spinach (*Ipomoea aquatica*: 21.29 ± 0.43 mg GAE/g) [55] in Bangladesh. Additionally, freeze-dried *Centella asiatica* leaves showed a lower TPC level (45.59 ± 3.04 mg GAE/g DW) [56]. Ultrasound-assisted extraction of phenolic compounds from spinach (Spinacea oleracea L.) also showed comparatively much lower TPC (33.96 mg GAE/g DW) than our studied unconventional leafy vegetables [57]. Moreover, a previous study on some leafy vegetables in Sri Lanka reported that the TPC ranged from 0.92 to 11.03 mg GAE/g DW, which is comparatively much lower than our studied leafy vegetables [58]. *Bambusa* spp. shoots also had a high amount of TPC (41.7 ± 3.09 mg GAE/g DW) (Fig. 3a). A high-performance liquid chromatography (HPLC) study on bamboo shoots identified and quantified eight phenolic compounds, including ferulic acid, p-coumaric acid, syringic acid, chlorogenic acid, caffeic acid, catechin, p-hydroxybenzoic acid, and protocatechuic acid [59].

However, the TPC was significantly lower in the seed samples viz., *P. speciosa* (5.797 ± 1.185 mg GAE/g DW) and *L. leucocephala* (3.877 ± 0.536 mg GAE/g DW). Previous studies reported comparatively higher TPC in the ethanolic extracts of *P. speciosa* seeds (14.90–26.3 mg GAE/g DW) in Malaysia [60] and *L. leucocephala* seeds (37.38 ± 0.49 mg GAE/g DW) in Thailand [36]. The location of sampling significantly affects the TPC of *P. speciosa* [60]. The TPC of *P. speciosa* seeds includes five phenolic compounds such as gallic acid, p-coumaric acid, trans-cinnamic acid, ferulic acid, and caffeic acid [60]. The TPC of these seeds was comparable to the commonly consumed pumpkin *(Cucurbita pepo)* seeds (4.28 ± 1.85 mg GAE/g DW) [61]. However, common beans (*Phaseolus vulgaris* L.), a widely consumed legume, had a lower TPC value (0.11–4.59 mg GAE/g DW) than the samples we examined [62].

### 3.2 Total flavonoid content

Fig. 3b represents the total flavonoid content in the methanolic extracts of the studied samples. The TFC was significantly higher in *the P. odorata* leaf compared to the other four vegetables studied. The flavonoid content of the 5 samples decreased in the following order: *P. odorata* leaf (9.19 ± 0.7 mg QE/g DW) > *N. arbor-tristis* leaf (5.04 ± 0.46 mg QE/g DW) > *Bambusa* spp. shoot (2.57 ± 0.494 mg QE/g DW) > *P. speciosa* seed (0.64 ± 0.16 mg QE/g DW) > *L. leucocephala* seed (0.58 ± 0.12 mg QE/g DW).

In this study, the TFC followed a similar trend to the TPC, being higher in leafy vegetables than in shoots and seeds (Fig. 3b). The highest value of TFC was observed in *P. odorata* leaf extract. However, a previous study reported a comparatively higher TFC value (19.97 mg QE/g DW) in *P. odorata* leaves than our findings [49]. Another study found a higher TFC value in this leaf extract (70.65 μg QE/mg DW) while using ethanol as a solvent [50]. An ultra-high performance liquid chromatography (UHPLC) analysis identified several flavonoid compounds in the *P. odorata* leaf extract, including (+)-catechin, quercetin 3-O-β-D-rhamnoside (quercitrin), quercetin 3-O-α-L-arabinopyranoside (guajavarin), quercetin 3-O-β-D-glucoside (isoquercitrin), and quercetin 3-O-β-D-galactopyranoside (hyperoside) in the *P. odorata* leaf extract [32]. In a prior investigation, the TFC of *N. arbor-tristis* leaf samples was determined to be 2.13–12.36 mg QE/g DW, which seems closer to our findings [38]. Several other studies revealed considerably greater values for TFC in ethanolic extracts of *N. arbor-tristis* leaves than in methanolic extracts [63,64]. The ethanolic solvent may become more effective at extracting flavonoid compounds because of their polar characteristics [50,65,66]. The studied seed samples, such as *P. speciosa* seed and *L. leucocephala* seed extract, had the lowest levels of TFC. However, the TFC of studied *P. odorata* was slightly higher than that of the Malabar spinach (8.68 mg/g DW) found in a previous study [67]. A previous study of Ghasemzadeh et al. reported that the TFC varied significantly in *P. speciosa* seed depending on the sampling sites and ranged from 7.4 to 12.4 mg QE/g DW [60]. An ultra-high-performance liquid chromatographic study on *P. speciosa* seed identified six distinct flavonoids such as quercetin, myricetin, luteolin, catechin, kaempferol, and rutin [60]. These findings are in line with earlier studies which found that the leaves typically contain more flavonoids than seeds, flowers, stems, or bark [[67], [68], [69]]. It may be due to the fact that leaves are generally exposed to more sunlight, which triggers an increase in flavonoid content [70]. Moreover, the synthesis of flavonoid compounds in plant samples depends on various factors like environmental conditions (CO_2_ concentration, temperature, precipitation), agricultural practices (irrigation, fertilization, harvesting, post-harvesting), and the location of the plantation [60]. The flavonoids have strong antioxidant potential and significantly impact human nutrition and health [4]. The studied vegetables may be used to treat rheumatic diseases, gastrointestinal ulcers, coronary heart disease, and cancer due to their high TFC values [71].

### 3.3 Total tannin content

The TTC of the samples was assessed and expressed as tannic acid equivalents (TAE). Among the samples, *P. odorata* leaf exhibited the highest TTC (3.59 ± 0.26 mg TAE/g DW), followed by *N. arbor-tristis* leaf (1.78 ± 0.08 mg TAE/g DW), *L. leucocephala* seed (1.17 ± 0.06 mg TAE/g DW), *P. speciosa* seed (0.87 ± 0.18 mg TAE/g DW), and *Bambusa* spp. shoot (0.67 ± 0.177 mg TAE/g DW) (Fig. 3c). A previous study reported comparatively higher TTC value in the *P. odorata* leaves (11.5 ± 0.50 mg TAE/g DW) cultivated in Malaysia [51]. A very little amount of TTC was found in our studied *P. speciosa* seed extracts. However, earlier research didn't find any tannin substances in *P. speciosa* cultivated in Malaysia [60]. The present study reveals that the TTC in leafy vegetables is comparatively higher than the studied seed and shoot-type vegetables, which may be due to the oxygen exposure leading to the formation of oxidized tannins [72]. The *Bambusa* spp. shoot extract had the lowest TTC, despite having moderate levels of TPC and TFC. Although the tannin content is lower than other bioactive components, its presence is significant as tannins can interfere with the absorption of nutrients in the gut [73].

### 3.4 DPPH scavenging activity

**Fig. 4:**
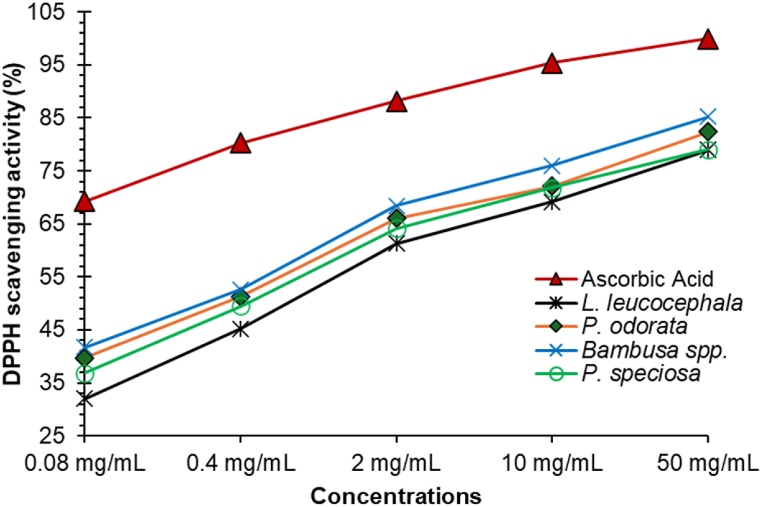
Comparative analysis of DPPH scavenging activity of *P. speciosa, L. leucocephala, P. odorata, N. arbor-tristis,* and *Bambusa* spp. with the standard ascorbic acid at different concentrations.

Antioxidant efficacy can be readily evaluated using DPPH radical scavenging, which is regarded as a reliable *in vitro* model [47]. Significant dose-dependent DPPH radical scavenging activity of the selected Manipuri vegetables was assessed, and the results are illustrated in Fig. 4. The antioxidant activity against DPPH radical was determined at different concentrations (0.08, 0.4, 2, 10, and 50 mg/mL) to get a standard graph whereas the ascorbic acid standard was used. The results show that DPPH scavenging activity increased with the concentration of the standard. The standard ascorbic acid exhibited a strong antioxidant activity (69.23 ± 1.6) even at a very low concentration (0.08 mg/mL).

All the selected sample extracts had potential DPPH scavenging activity which was increased with the concentration of each plant extract. In concentrations ranging from 0.08 to 50 mg/mL, *P. speciosa* exhibited DPPH scavenging activity that varied between 36.83 ± 2.15 % and 79.01 ± 1.15 %. Similarly, *L. leucocephala* showed activity from 32.05 ± 0.68 % to 78.96 ± 1.85 %, *P. odorata* from 39.69 ± 2.18 % to 82.38 ± 3.12 %, *N. arbor-tristis* from 43.57 ± 1.12 % to 81 ± 2.32 %, and *Bambusa* spp. from 41.69 ± 1.32 % to 85.26 ± 2.15 % (Fig. 4). This result showed that *P. odorata* leaf and *Bambusa* spp. shoot extract had the strongest scavenging activity at 50 mg/mL concentration**.** Furthermore, at a low concentration of 0.08 mg/mL, *N. arbor-tristis* leaf extracts exerted the highest scavenging activity, while *L. leucocephala* seed extract exhibited the lowest value.

**Fig. 5:**
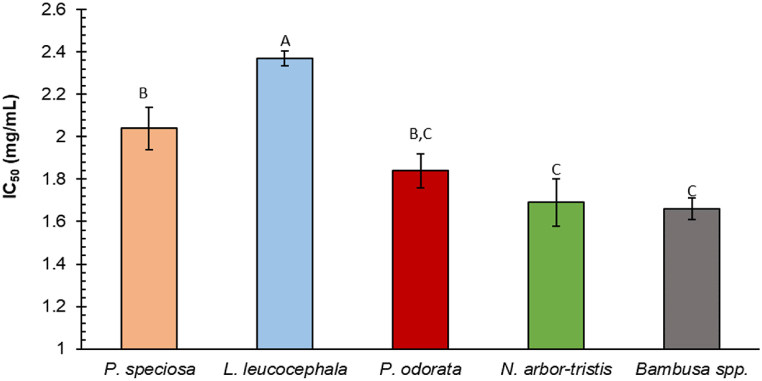
Antioxidant activity of five commonly consumed Manipuri vegetable samples. Results represent means ± standard deviation (n = 3). Different letters in the column indicate the significant differences among the samples (Tukey's test, p < 0.05).

The concentration of the sample required to block 50 % of DPPH radicals is known as the IC_50_, and lower values indicate better antioxidant capacity [74]. Fig. 5 shows the IC_50_ value representing the antioxidant property of the selected sample extracts.

According to IC_50_ value, *Bambusa* spp. shoot had the strongest DPPH radical scavenging activity (1.66 ± 0.05 mg/mL), followed by *N. arbor-tristis* leaf (1.69 ± 0.11 mg/mL), *P. odorata* leaf (1.84 ± 0.08 mg/mL), *P. speciosa* seed (2.04 ± 0.10 mg/mL), and *L. leucocephala* seed (2.37 ± 0.034 mg/mL) extract.

From the earlier results, it was found that *P. odorata* had relatively higher phenolic compounds, flavonoids, and tannins. However, *Bambusa* spp. has considerably greater antioxidant activity, which might be attributed to the presence of many bioactive substances such as polyphenols, and vitamins A, C, and E. A previous study on *Phyllostachys pubescence* shoots also identified eight phenolic acids*,* such as p-coumaric acid, ferulic acid, syringic acid, chlorogenic acid, caffeic acid, catechin, protocatechuic acid, and p-hydroxybenzoic acid [59,75]. Moreover, it contains selenium in trace amounts which exhibits potential antioxidant activity [59]. Ascorbic acid content in the methanolic extract of bamboo shoots such as *Phyllostachys nigra* (195.3 ± 3.4 mg/100 g DW) and *Phyllostachys pubescens* (154.7 ± 8.5 mg/100 g DW) contributes to the antioxidant capacity [59]. Bamboo shoots are being promoted as a healthy food for their nutritional value and antioxidant-rich bioactive compounds. A previous study reported comparable DPPH radical scavenging activity for the methanolic extracts of bamboo shoots (IC_50_: 3.6 and 3.4 mg/mL) [59]. A study by Kong et al. also found that all extracts from young *Bambusa vulgaris* shoots demonstrated strong DPPH radical scavenging activity, which aligns with our results [76].

*N. arbor-tristis* and *P. odorata* leaf extracts also exhibited good antioxidant properties (Fig. 4). An earlier study found two potential antioxidant compounds, such as n-hexadecanoic acid and cis-9-hexadecenal, in the *N. arbor-tristis* through GCMS analysis and TLC bioautography [38]. UHPLC study reported that the *P. odorata* leaf contained methyl gallate, (+)-catechin, kaempferol sulfate, quercetin sulfate, tetrahydroxyflavonol derivative, quercetin 3-O-β-D-rhamnoside, quercetin 3-O-β-D-glucuronide, (epi)catechin gallate, and sinapic acid hexoside, which are attributed to its antioxidant potential [32]. A previous study reported high IC_50_ values for some Indian green leafy vegetables, such as *Amaranthus* sp. (IC_50_: 27.27 mg/mL), *Centella asiatica* (IC_50_: 19.89 mg/mL), *Trigonella foenum graecum* (IC_50_: 27.69 mg/mL), and *Murraya koenigii* (IC_50_: 9.62 mg/mL) [52], indicating their lower antioxidant potential compared to our studied samples. *L. leucocephala* seed extract had the lowest antioxidant capacity. However, it is comparable to the antioxidant potential of pumpkin seeds (IC_50_: 1.74 ± 0.24 mg/mL) [61].

This study reveals that the antioxidant capacity was significantly higher in the leafy vegetables than in the seeds, which aligns with their phenolic compounds, flavonoids, and tannins. Many flavonoids and related polyphenols have been demonstrated in recent research to significantly increase the overall antioxidant activity of various fruits and vegetables [69,77,78]. The redox properties of phenolic compounds, which allow them to operate as hydroxy radical quenchers, proton donors, electron transfer agents, metal ion chelators, and singlet oxygen inhibitors, are the leading cause of their antioxidant action [79]. The majority of human illnesses, such as cancer and cardiovascular disease, are linked to higher levels of free radical production. Thus, dietary antioxidants found in the vegetables tested may have an important role in safeguarding proteins, lipids, and cellular DNA from free radical damage, making them crucial in the prevention of diseases [52].

### 3.5 Antibacterial activity of the food plant extracts

**Fig. 6:**
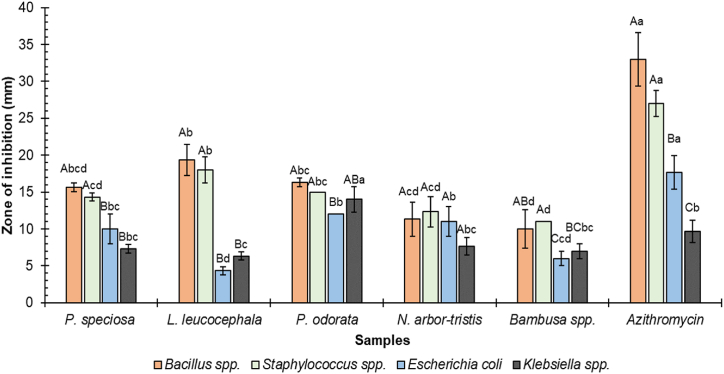
Zone of inhibition (ZOI) exerted by the samples against bacteria. Results represent means ± standard deviation (n = 3). Different capital letters indicate significant differences in the ZOI within the same sample for different bacterial species, and different lowercase letters indicate significant differences in the ZOI for the same bacterial species among different samples.

Antibacterial activities for methanolic extracts of selected plant samples were examined against 2 g-positive (viz., *Bacillus* spp.*, and Staphylococcus* spp.) and 2 g-negative bacteria (viz., *Escherichia coli,* and *Klebsiella* spp.). Fig. 6 Represents the exerted zone of inhibition by the selected sample extracts against the studied bacteria.

Among the tested vegetables, *L. leucocephala* seed extract showed the highest antibacterial activity against gram-positive bacteria, *Bacillus* spp. (ZOI: 19.33 ± 2.08 mm) and *Staphylococcus* spp. (ZOI: 18 ± 1.73 mm), while it showed the lowest activity against gram-negative bacteria, *Escherichia coli* (ZOI: 4.33 ± 0.578) and *Klebsiella* spp*.* (ZOI: 6.33 ± 0.58). *P. odorata* leaf extract also exerted a good antibacterial potency against both the tested gram-positive and gram-negative bacteria, whereas the ZOI ranges from 12 to 16.33 ± 0.58 mm. *Bambusa* spp. shoot extract exhibited comparatively lower antibacterial activity against all the tested pathogens.

**Fig. 7:**
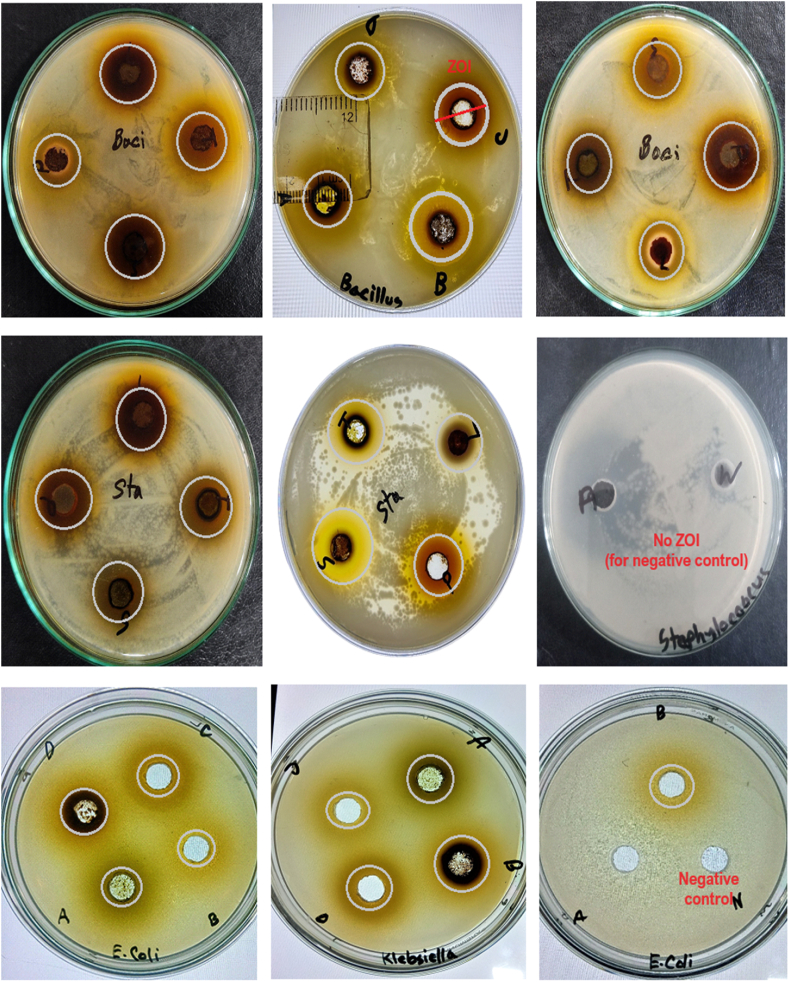
Some representative pictures of Petri dishes showing zones of inhibition.

Moreover, azithromycin was employed as a positive control for measuring the ZOI against the selected bacterial strains, which exhibited a high antibacterial potency against gram-positive bacteria *Bacillus* spp. (ZOI: 33 ± 3.06 mm)*, and Staphylococcus* spp. (ZOI: 27 ± 1.73 mm). In contrast, a comparatively lower ZOI was observed for the *Klebsiella* spp. (9.66 ± 1.52 mm). *P. speciosa* seed extract also had similar effects on the tested microorganisms, where the ZOI value ranged from 7.33 to 15.66 mm. According to a previous study, *P. speciosa* pod extract showed antibacterial potency with ZOI ranging from 6.87 to 11.50 mm against *E. coli, Staphylococcus aureus, Listeria monocytogenes,* and *Bacillus cereus* [80]. Another study found that *P. speciosa* seed extracts are more effective against gram-positive bacteria than gram-negative one [60], which is consistent with our findings. Fig. 7 represents some Petri dishes testing zones of inhibition exhibited by the studied sample extracts and controls.

The antibacterial properties of plant foods are mainly due to their secondary metabolites [81]. These phytochemicals can serve as alternative or supplementary treatments to antibiotics, either alone or in combination, to address the growing issue of antibiotic resistance [38]. Studies have previously documented the antibacterial and anti-inflammatory properties of n-hexadecanoic acid and cis-9-hexadecenal, which are present in the leaves of *N. arbor-tristis* [38,81]. The antibacterial potency of *L. leucocephala* seeds may be attributed to the presence of several active ingredients, such as kaempferol, myricetin, naringenin, rosmarinic acid, quercetin, resveratrol, o-coumaric acid, ellagic acid, rutin, ferulic acid, benzoic acid, p-coumaric acid, caffeic acid, chlorogenic acid, catechin, p-hydroxybenzoic acid, and catechol, which were found in methanolic extracts from the seeds obtained through phytochemical analysis using HPLC [82].

### 3.6 Minimum inhibitory concentration (MIC) and minimum bactericidal concentration (MBC) of the food plant extracts

The MIC and MBC of *P. speciosa* seed (S1), *L. leucocephala* seed (S2), *P. odorata* leaf (S3), *N. arbor-tristis* leaf (S4), and *Bambusa* spp. shoot (S5) extracts against gram-positive bacteria (*Bacillus* spp. and *Staphylococcus* spp.) and gram-negative bacteria (*Escherichia coli* and *Klebsiella* spp.) were determined. The MIC value is defined as the lowest concentration of extract that inhibits the growth of specific bacteria, whereas the MBC value is the lowest concentration that can kill any tested pathogen [42]. The MIC values varied among the selected sample extracts (Table 1).

**Table 1:** Determination of MIC value of sample extracts against bacteria by nutrient broth dilution method.

Bacterial species	Concentration of samples																									MIC (mg/mL)				
	125 mg/mL					62.5 mg/mL					31.25 mg/mL					15.6 mg/mL					7.8 mg/mL									
	S1	S2	S3	S4	S5	S1	S2	S3	S4	S5	S1	S2	S3	S4	S5	S1	S2	S3	S4	S5	S1	S2	S3	S4	S5	S1	S2	S3	S4	S5
*Bacillus* sp.	–	–	–	–	–	–	–	–	–	–	–	–	–	+	+	–	–	+	+	+	+	+	+	+	+	15.6	15.6	31.25	62.5	62.5
*Staphylococcus* sp.	–	–	–	–	–	–	–	–	–	–	–	–	–	+	+	+	–	+	+	+	+	+	+	+	+	31.25	15.6	31.25	62.5	62.5
*E. coli*	–	–	–	–	–	–	+	–	–	+	+	+	+	+	+	+	+	+	+	+	+	+	+	+	+	62.5	125	62.5	62.5	125
*Klebsiella* sp.	–	–	–	–	–	+	+	–	+	+	+	+	–	+	+	+	+	+	+	+	+	+	+	+	+	125	125	31.25	125	125

*Here, (−) means no visible growth of bacteria, (+) denotes the visible growth; *S1 = *P*. *speciosa*, S2 = *L*. *leucocephala,* S3 = *P*. *odorata,* S4 = *N*. *arbor-tristis*, S5 = *Bambusa* spp.

The most potent antibacterial agent for gram-positive bacteria (*Bacillus* spp. and *Staphylococcus* spp.) was *L. leucocephala* with a MIC value of 15.6 mg/mL. Among gram-negative bacteria, *P. odorata* had the lowest MIC value for *Klebsiella* spp. (31.25 mg/mL), while *P. speciosa, P. odorata,* and *N. arbor-tristis* had the lowest MIC value for *E. coli* (62.5 mg/mL). *Klebsiella* spp. showed the highest resistance to the plant extracts, except for *P. odorata*. The plant extracts had higher MIC values against gram-negative bacteria. The least effective plant extract was *Bambusa* spp., with the highest MIC values against all tested bacterial strains.

The MBC values of the food plant extracts were equal to or higher than the MIC values (Table 2).

**Table 2:** Determination of MBC value of sample extracts against bacteria.

Bacterial species	Concentration of samples																									*MBC (mg/mL)*				
	125 mg/mL					62.5 mg/mL					31.25 mg/mL					15.6 mg/mL					7.8 mg/mL									
	S1	S2	S3	S4	S5	S1	S2	S3	S4	S5	S1	S2	S3	S4	S5	S1	S2	S3	S4	S5	S1	S2	S3	S4	S5	S1	S2	S3	S4	S5
*Bacillus* sp.	–	–	–	–	–	–	–	–	–	–	–	–	–	+	+	+	+	+	+	+	+	+	+	+	+	31.25	31.25	31.25	62.5	62.5
*Staphylococcus* sp.	–	–	–	–	–	–	–	–	–	+	–	–	+	+	+	+	+	+	+	+	+	+	+	+	+	31.25	31.25	62.5	62.5	125
*E. coli*	–	–	–	–	–	–	+	–	–	+	+	+	+	+	+	+	+	+	+	+	+	+	+	+	+	62.5	125	62.5	62.5	125
*Klebsiella* sp.	–	–	–	–	+	+	+	–	+	+	+	+	–	+	+	+	+	+	+	+	+	+	+	+	+	125	125	31.25	125	–

*Here, (−) means no visible growth of bacteria, (+) denotes the visible growth; *S1 = *P*. *speciosa*, S2 = *L*. *leucocephala*, S3 = *P*. *odorata*, S4 = *N*. *arbor-tristis*, S5 = *Bambusa* spp.

*P. speciosa* and *L. leucocephala* seed extracts showed the most bactericidal activity against gram-positive bacteria (*Bacillus* spp. and *Staphylococcus* spp.), with an MBC value of 31.25 mg/mL. The lowest MBC value for *Escherichia coli* was found in *P. speciosa, P. odorata,* and *N. arbor-tristis* extracts (62.5 mg/mL), while the lowest MBC value for *Klebsiella* spp. was found in *P. odorata* extracts (31.25 mg/mL). The MBC values for gram-negative bacteria are similar to the MIC values. Bambusa spp. could not kill Klebsiella spp. even at a concentration of 125 mg/mL, suggesting it was the least effective.

The antibacterial activity of the indigenous food plants revealed that all the plant extracts were more effective against gram-positive bacteria (*Bacillus* spp. and *Staphylococcus* spp.) with lower MIC and MBC values compared to gram-negative bacteria (*Escherichia coli* and *Klebsiella* spp.). Plant extracts usually carry polyphenols which show antimicrobial activity [83]. Previous research reported that polyphenols and antibiotics also show better efficacy against gram-positive bacteria than gram-negative bacteria [84,85]. The antibacterial activity of the tested sample extracts, except for *P. odorata.,* was comparatively lower against the *Klebsiella* spp. It may be due to the structural differences in bacterial cells [86]. Gram-negative bacteria have a thinner cell wall surrounded by a thick lipopolysaccharide outer membrane, making it more difficult for antimicrobial agents to penetrate [87]. Moreover, multidrug efflux pumps in their cell membranes may contribute to their lower sensitivity to antimicrobials [88,89]. *L. leucocephala* seed extract exhibited a lower MIC for the tested gram-positive bacteria while showing a higher MIC for the tested gram-negative bacteria. However, the *P. odorata* leaf extract exhibited the highest antibacterial activity against the tested gram-negative bacteria with the lowest and equal MIC and MBC values (*Klebsiella* spp.: 31.25 mg/mL and *E. coli*: 62.5 mg/mL). This antibacterial potency aligns with the polyphenols, flavonoids, and tannin contents of the *P. odorata* leaf. Moreover, decanal, caryophyllene, and dodecanal are present in the *P. odorata* leaf extract, which may be responsible for antibacterial activity [90]. The *P. speciosa* and *L. leucocephala* seed extracts showed the most bactericidal activity against gram-positive bacteria (MBC: 31.25 mg/mL). The *P. speciosa* seed extract contains hexathionine and trithiolane [[91], [92], [93]], which may trigger its antimicrobial potency [94,95]. The *Bambusa* spp. shoot extract exhibited comparatively lower antibacterial activity against all the tested pathogens, even at a high concentration of 125 mg/mL. Though, in some cases, our selective plant extracts appeared as ineffective antimicrobials, the reasons may not be for plant characteristics; rather, variations in microbial habitat, inherent resistance, and mutation in bacterial strain may be the causes [96]. However, the tested samples had potential antimicrobial properties, which may replace synthetic antimicrobials in food and pharmaceutical industries.

This is pioneering research on the specific Manipuri vegetables evaluating their antioxidant and antibacterial efficacy. Although this study has numerous merits, it also has some limitations. The antioxidant compounds of vegetable samples may vary according to the species, variety, soil characteristics of the cultivation, seasons, climatic conditions, and extraction conditions. We did not consider any of those factors. Moreover, advanced analyses for phenolic profiling are recommended for these samples, which may provide a more comprehensive understanding of the antioxidant and antimicrobial properties.

## 4 Conclusion

The present study highlights the significant phytochemical content, particularly phenolics, flavonoids, and tannins in selected indigenous Manipuri vegetables, demonstrating their remarkable antioxidant and antibacterial properties. Based on this study, among the five samples, the leafy vegetables (viz., *P. odorata*, *N. arbor-tristis*) had comparatively higher antioxidant compounds, including TPC, TFC, and TTC. However, the bamboo shoot exhibited the highest antioxidant potential. The examined gram-positive bacterial strains showed comparatively more sensitivity to the sample extracts than the gram-negative ones. This study also found the potency of *P. odorata* leaf extract against gram-negative bacteria. These findings suggest that these vegetables could be valuable sources of antioxidant and antibacterial compounds that can contribute to alternative drug discovery and nutritional strategies. Additional research is required to assess the other nutritional and antinutritional properties of these underutilized vegetables to suggest them in the diet list of the population in Bangladesh.

## Data availability statement

Data will be made available on request.

## CRediT authorship contribution statement

**Mukta Roy:** Writing – review & editing, Supervision, Project administration, Investigation, Funding acquisition, Data curation, Conceptualization. **Jahid Hasan Shourove:** Writing – original draft, Supervision, Project administration, Investigation, Data curation, Conceptualization. **Rhythm Singha:** Methodology, Investigation, Formal analysis, Data curation. **Tawkir Ahmed Tonmoy:** Methodology, Investigation, Formal analysis, Data curation. **Gokul Chandra Biswas:** Writing – review & editing, Supervision, Investigation, Data curation, Conceptualization. **Fariha Chowdhury Meem:** Writing – original draft, Methodology, Investigation, Data curation. **Parvej Hasan John:** Writing – original draft, Methodology, Investigation, Data curation. **Mitu Samadder:** Writing – review & editing, Visualization. **Md. Azmain Al Faik:** Writing – review & editing, Visualization.

## Declaration of competing interest

The authors declare that they have no known competing financial interests or personal relationships that could have appeared to influence the work reported in this paper.
